# Risk factors for underlying bilateral vestibular weakness in cochlear implant candidates

**DOI:** 10.1097/ONO.0000000000000066

**Published:** 2025-03-24

**Authors:** Allison Reeder, Joseph Canner, Nofrat Schwartz

**Affiliations:** 1 Yale University School of Medicine, New Haven, CT; 2 Division of Otolaryngology-Head and Neck Surgery, Department of Surgery, Yale University School of Medicine, New Haven, CT.

**Keywords:** Cochlear implantation, Bilateral vestibular weakness, Videonystagmography, Vestibular weakness

## Abstract

**Introduction:**

Cochlear implantation (CI) is associated with postoperative vestibular dysfunction in the implanted ear; however, limited data on baseline vestibular function in these patients exists. Bilateral vestibular weakness is associated with detrimental effects on quality of life. As such, it is important to identify patients with a preexisting bilateral weakness and consider this information in surgical planning.

**Methods:**

Retrospective cohort study of the CI candidate population. All patients underwent routine preoperative vestibular evaluation, irrespective of symptoms.

**Results:**

Of 180 preoperative videonystagmographies, 39.4% showed vestibular weakness determined by caloric testing. Of these, 26.8% exhibited bilateral weakness. Patients with bilateral weakness had higher body mass index (31.6 kg/m^2^) than those with unilateral weakness or normal function (26.2 and 27.4 kg/m^2^, *P* = 0.007). Further analysis of the audiologic data in the worse-hearing ear revealed worse hearing at 250, 500, and 1000 Hz (*P* < 0.05). Hearing threshold of 60 dB or worse at 250 Hz was the best prognostic indicator for bilateral weakness. At a threshold of 60 dB at 250 Hz, all patients with bilateral weakness are captured (100% sensitivity), with a specificity of 34.5%.

**Conclusion:**

More than one-third of CI candidates have some degree of underlying vestibular dysfunction and 10.5% exhibit preexisting bilateral weakness. This study indicates that audiologic data may be a useful prognostic indicator of preexisting bilateral vestibular weakness. Given the well-documented detrimental effects of bilateral vestibular weakness on quality of life, we recommend that all patients who meet this cutoff undergo vestibular testing to assess for an underlying weakness.

Cochlear implantation (CI) has been associated with postoperative vestibular dysfunction in the implanted ear (1–5). Advances in surgical approach and electrode types have allowed for less traumatic implantation with better preservation of neural elements and hearing. Despite this, there has been concern that manipulation of the inner ear and placement of a foreign body can cause significant inflammation and possible damage to the vestibular system (1,6). There is limited data on preoperative vestibular function in this population and risk factors for preexisting dysfunction are not well documented in the existing literature.

Traditionally, in a preoperative CI evaluation, the audiologic data is the driving factor for surgical planning of which ear to implant. Although iatrogenic postoperative vestibular dysfunction is a known risk of this procedure, preoperative evaluation of the vestibular system is not common practice and thus not routinely considered in preoperative decision-making and counseling. It is reasonable to hypothesize that patients with preexisting vestibular dysfunction, particularly those with weakness bilaterally, may be at higher risk for increased morbidity as a result of implantation. Studies have reported that bilateral vestibular weakness is associated with detrimental effects on quality of life, including decreased social engagement and difficulty performing activities of daily living (7,8). As such, it is important to identify patients with preexisting subclinical bilateral weakness who may be at an elevated risk for the development of symptomatic vestibular dysfunction postoperatively.

This study aims to evaluate the prevalence of underlying bilateral vestibular weakness in the traditional cochlear implant candidate population with moderate to severe hearing loss. We further aim to identify demographic and audiologic characteristics that may be associated with higher risk for preexisting bilateral vestibular dysfunction to assist in the identification of this condition and aid in preoperative surgical decision-making and counseling.

## METHODS

### Patients

We conducted a retrospective cohort study of the traditional CI candidate population evaluated at a single institution over 10 years (2012–2022). The patients included in this data set were determined by electronic medical record query for patients who underwent CI over the last 10 years (CPT 69930), using the Joint Data Analytics Team (JDAT), part of the Yale Center for Clinical Investigation/Yale School of Medicine. This method of determining our patient population was selected only for those cochlear implant candidates who underwent surgical implantation. Patients who were cochlear implant candidates but did not elect to undergo surgery, for any reason, were not captured and thus not able to be included in our analyses. Demographic data obtained from the JDAT database included age, gender, ethnicity, and comorbidities. Chart review was conducted to record videonystagmography (VNG) and audiologic data. All patients evaluated for CI underwent routine preoperative vestibular evaluation as part of CI evaluation, irrespective of symptoms. This was considered routine, standard of care, in the specific practice for all CI candidates. Patients found to have bilateral preoperative vestibular weakness were included for further analysis.

### Screening for Cochlear Implant Candidacy

All patients included in this study were determined to be cochlear implant candidates and elected to pursue and undergo CI. Patients with severe to profound sensorineural hearing loss in both ears, limited benefit from hearing aids demonstrated by poor speech recognition scores (typically below 50% correct in the ear to be implanted and below 60% in the best-aided condition), and a willingness to participate in extensive rehabilitation therapy were found to be candidates. We utilize the AzBio Sentence Test and Monosyllabic Word Test (consonant-nucleus-consonant word test) to determine candidacy for a cochlear implant. Testing is completed in the sound field at a presentation level of 50 dB HL using both hearing aids.

### Audiologic Data

Audiologic data from the patient’s preoperative CI candidacy evaluation was obtained via chart review. Pure tone thresholds at 250, 500, 1000, 2000, 4000, and 8000 Hz were recorded for both ears. Pure tone average (PTA) (mean of 500, 1000, and 2000 Hz) and low pure tone average (LPTA) (mean of 250, 500 Hz) were calculated manually. Word recognition score (WRS), the percentage of single syllable words correctly repeated by a patient when presented at 40 dB above the speech recognition threshold, was also obtained.

### Videonystagmography

Vestibular function was tested by comprehensive preoperative VNG, irrespective of symptoms, in all patients determined to be cochlear implant candidates as described above. The specific test of interest for this study was caloric testing, which is used to investigate the function of the horizontal semicircular canal. Bilateral ears are individually stimulated with cool and/or warm air. VNG is then used to monitor/record horizontal eye movements during these stimulations. Patients were classified as either having a “weak” or “normal” vestibular response for each ear based on the results of caloric testing. Percentage of vestibular function was not reliably recorded for all patients and thus was not included for the analysis. We have recommended future studies to address the quantitative as well as the qualitative components of the vestibular weakness. To analyze the responses, the “total” response for each ear is calculated. The value used was the maximum slow phase velocity (SPV). For example, the total response for the right ear is defined as: peak (in SPV) for cool response minus peak warm response (Total right ear response [RE] = Peak right cold caloric response − Peak right warm caloric response). Total response for the left ear is the same, (Total left ear response [LE] = Peak left warm caloric response − Peak left cold caloric response). Unilateral weakness (UW) is calculated by taking the ([TotalRE – TotalLE] divided by [TotalRE + TotalLE]) × 100. This gives the UW percentage. To classify as having UW, the percentage is typically (may vary by clinic) 25% or greater. This indicates that the patient has a weakness of xx% in the calculated ear. If the percentage is less than 25%, the patient is classified as having “normal” peripheral function. Although the exact number may vary based on clinic protocol, bilateral weakness is traditionally based on combined caloric responses for both ears. Therefore, when looking at the TotalRE + TotalLE, a bilateral weakness is defined as 24 degrees/second or less (9).

### Statistical Analysis

To determine variables associated with vestibular weakness, we compared the means of continuous variables between those with and without vestibular weakness using a Student’s *T* test and we compared proportions of categorical variables using a chi-square test. As appropriate, we constructed logistic regression models with vestibular weakness as the dependent variable and variables with significant associations with vestibular weakness as the independent variables.

## RESULTS

**FIG. 1.**
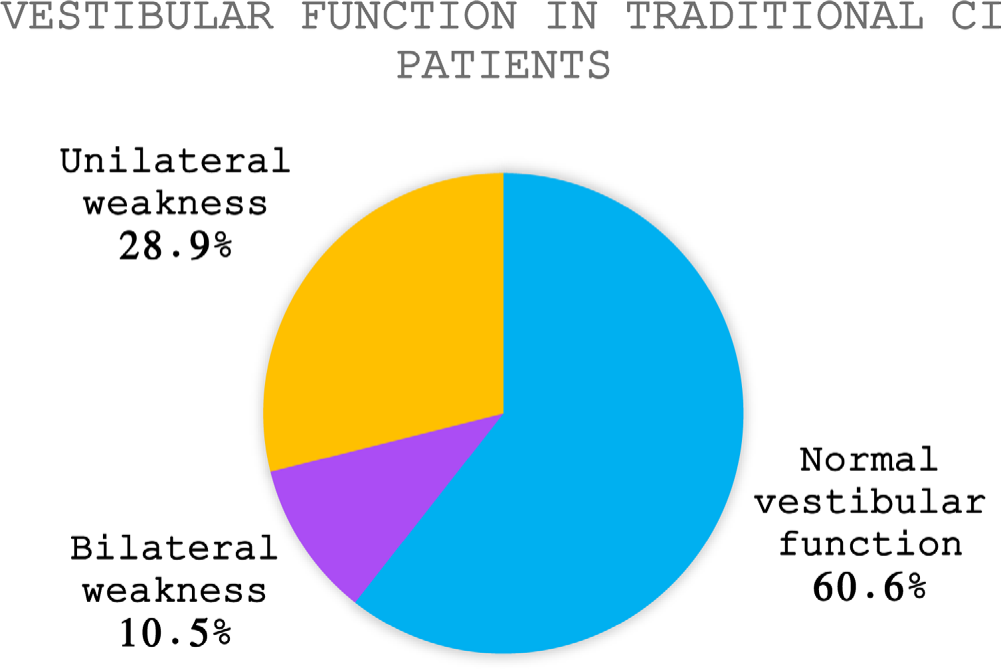
Vestibular status of traditional cochlear implant candidates.

A total of 180 preoperative VNGs were obtained over the study period from 2012 to 2022. Of this cohort, 39.4% demonstrated evidence of vestibular weakness as determined by caloric testing irrespective of symptoms. Of these, 26.8% exhibited a bilateral weakness (Fig. 1). No comorbidities, including history of myocardial infarction, peripheral vascular disease, or diabetes were found to be associated with a bilateral weakness. Patients with bilateral weakness had higher body mass index (BMI) (31.6 kg/m^2^) than those with UW or normal function (26.2 and 27.4 kg/m^2^, *P* = 0.007). Age approached significance with bilaterally weak patients tending to be younger (58.9 years) compared to UW or normal function (68.1 vs 66.3 years, *P* = 0.089). Gender was not found to correlate with bilateral weakness with 55.7% of bilaterally weak patients being female compared to 51% of unilaterally weak and 59.4% of normal functioning patients (*P* = 0.455) (Table 1).

**TABLE 1.** Demographic characteristics and comorbidities of cochlear implant candidates

Demographic and clinical characteristics of cochlear implant candidates							
	Weakness						
	Normal		Unilateral		Bilateral		*P*
Number of patients, N (% of total patients)	106.	(100.0)	51.	(100.0)	17.	(100.0)	
Patient gender, N (%)							
Female	63	(59.4)	26	(51.0)	8	(47.1)	0.455
Male	43	(40.6)	25	(49.0)	9	(52.9)	
Age (years)							
Mean (SD)	66.3	(15.0)	68.1	(13.7)	58.9	(16.8)	0.089
Median (IQR)	70	(18)	71	(18)	60	(14)	0.085
Patient BMI (kg/m^2^)							
Mean (SD)	27.4	(5.9)	26.2	(3.9)	31.6	(7.1)	0.007
Median (IQR)	26	(7)	27	(5)	32	(12)	0.044
History of MI, N (%)							
No	98	(92.5)	49	(96.1)	15	(88.2)	
Yes	8	(7.5)	2	(3.9)	2	(11.8)	0.497
History of CVA, N (%)							
No	84	(79.2)	41	(80.4)	15	(88.2)	
Yes	22	(20.8)	10	(19.6)	2	(11.8)	0.686
History of pulmonary disease, N (%)							
No	77	(72.6)	34	(66.7)	13	(76.5)	
Yes	29	(27.4)	17	(33.3)	4	(23.5)	0.654
Diabetes, N (%)							
No	89	(84.0)	38	(74.5)	15	(88.2)	
Yes	17	(16.0)	13	(25.5)	2	(11.8)	0.272
History of chronic kidney disease, N (%)							
No	92	(86.8)	42	(82.4)	15	(88.2)	
Yes	14	(13.2)	9	(17.6)	2	(11.8)	0.721
History of hypertension, N (%)							
No	42	(39.6)	24	(47.1)	7	(41.2)	
Yes	64	(60.4)	27	(52.9)	10	(58.8)	0.675
History of hyperlipidemia, N (%)							
No	64	(60.4)	29	(56.9)	8	(47.1)	
Yes	42	(39.6)	22	(43.1)	9	(52.9)	0.574
History of coronary artery disease, N (%)							
No	83	(78.3)	37	(72.5)	15	(88.2)	
Yes	23	(21.7)	14	(27.5)	2	(11.8)	0.390

BMI indicates body mass index; CVA, cerebrovascular accident; IQR, interquartile range; MI, myocardial infarction; SD, standard deviation.

The audiologic data utilized for further analysis of patients with bilateral vestibular dysfunction were completed for the worse hearing ear. Analysis of the audiologic data in the worse hearing ear revealed worse hearing at 250, 500, and 1000 Hz in patients with bilateral vestibular weakness (*P* < 0.05). Patients with bilateral vestibular weakness had a mean hearing threshold of 88.5 dB at 250 Hz compared to 77 dB and 64.4 dB for unilaterally weak and normal functioning patients, respectively (*P* < 0.001). At 500 Hz, patients with bilateral weakness had a mean hearing threshold of 92.1 dB compared to 84.4 dB and 73.4 dB for unilaterally weak and normal functioning patients respectively (*P* < 0.001). At 1000 Hz, patients with bilateral weakness had a mean hearing threshold of 97.9 dB compared to 87.0 dB and 83.6 dB for unilaterally weak and normal functioning patients, respectively (*P* = 0.024) (Table 2).

**TABLE 2.** Preoperative audiologic data in cochlear implant candidates

Audiologic findings of cochlear implant candidates							
	Weakness						
	Normal		Unilateral		Bilateral		*P*
Hearing at 250 Hz (dB)							
Mean (SD)	64.4	(24.0)	77.0	(24.5)	88.5	(14.7)	0.000
Median (IQR)	65	(35)	75	(40)	85	(20)	0.000
Hearing at 500 Hz (dB)							
Mean (SD)	73.4	(23.8)	84.4	(24.9)	92.1	(14.8)	0.001
Median (IQR)	75	(35)	90	(40)	95	(25)	0.002
Hearing at 1000 Hz (dB)							
Mean (SD)	83.6	(21.0)	87.0	(19.5)	97.9	(14.4)	0.024
Median (IQR)	80	(30)	85	(35)	95	(20)	0.021
Hearing at 2000 Hz (dB)							
Mean (SD)	92.2	(19.3)	88.7	(20.1)	94.7	(16.1)	0.435
Median (IQR)	95	(30)	90	(35)	95	(20)	0.477
Hearing at 4000 Hz (dB)							
Mean (SD)	106.4	(100.7)	95.8	(19.9)	97.9	(13.0)	0.715
Median (IQR)	105	(30)	100	(35)	95	(10)	0.946
Hearing at 8000 Hz (dB)							
Mean (SD)	94.2	(12.9)	91.8	(14.1)	96.8	(10.1)	0.330
Median (IQR)	95	(10)	95	(15)	95	(10)	0.430
LPTA							
Mean (SD)	68.9	(23.0)	80.7	(24.2)	90.3	(14.3)	0.000
Median (IQR)	69	(35)	83	(38)	90	(23)	0.000
PTA							
Mean (SD)	83.1	(18.3)	86.7	(19.9)	94.9	(13.1)	0.042
Median (IQR)	82	(25)	85	(37)	92	(17)	0.034
WRS							
Mean (SD)	0.2	(0.2)	0.2	-0.2	0.2	(0.1)	0.410
Median (IQR)	0	(0)	0	(0)	0	(0)	0.310

IQR indicates interquartile range; LPTA, low pure tone average; PTA, pure tone average; SD, standard deviation; WRS, word recognition score.

The PTA and LPTA were also analyzed and found to be significantly lower in patients with bilateral vestibular weakness. Bilaterally weak patients were found to have a lower LPTA of 90.3 dB compared to 80.7 dB and 68.9 dB for unilaterally weak and normal functioning patients, respectively (*P* < 0.001). These patients were also found to have a lower PTA of 94.9 dB compared to 86.7 dB and 83.1 dB for unilaterally weak and normal functioning patients, respectively (*P* = 0.042) (Table 2).

WRS was not found to significantly correlate with vestibular function. Bilaterally weak patients were found to have a mean WRS of 15.1% compared to 17.7% and 21.2% for unilaterally weak and normal functioning patients, respectively (*P* = 0.410) (Table 2).

**FIG. 2.**
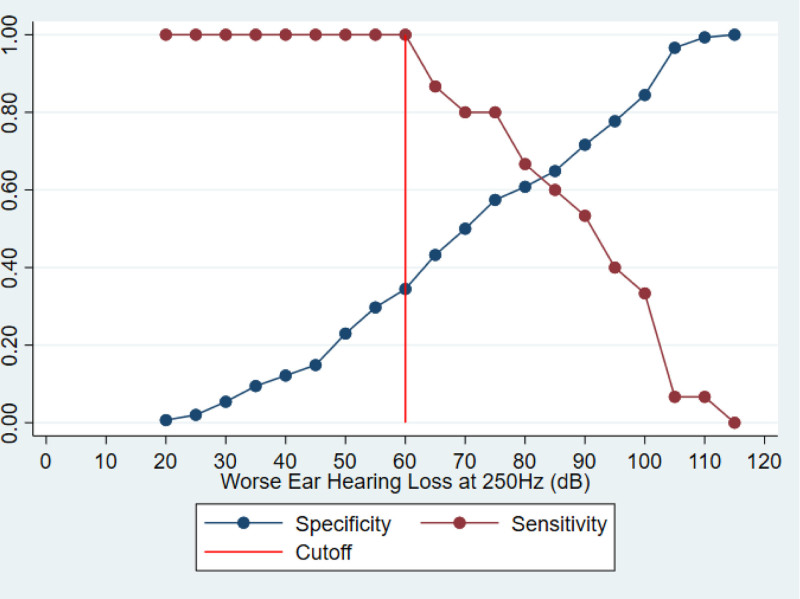
Sensitivity versus specificity across pure tone thresholds at 250 Hz in cochlear implant candidates. The y-axis represents sensitivity/ specificity (percentage) and the x-axis represents each pure tone threshold (dB) at 250 Hz. As specificity increases (blue points), sensitivity begins to decline (red points). 60 dB (red line) maximizes specificity while maintaining 100% sensitivity.

Further analysis was performed to try and identify an audiologic indicator for patients who may be at higher risk for baseline vestibular weakness. For practices that do not routinely perform vestibular testing before CI, we explored an audiologic indicator that would indicate the need for further vestibular testing in light of a higher risk of underlying bilateral vestibular weakness. A hearing threshold of 60 dB or worse at 250 Hz was found to be the best prognostic indicator for underlying bilateral weakness. At a threshold of 60 dB at 250 Hz, all patients with bilateral weakness are captured (100% sensitivity), with a specificity of 34.5%. After a threshold of 60 dB, sensitivity begins to decline. Using a cutoff of 60 dB, the specificity is maximized at 34.5% while maintaining 100% sensitivity (Fig. 2).

## DISCUSSION

CI is a common surgical technique for hearing restoration in adult patients with moderate to profound sensorineural hearing loss. Postoperative dizziness/disequilibrium has been cited as the most common complication following this procedure (10). Although the literature has described vestibular dysfunction as a possible complication of CI, it is not common practice to obtain routine preoperative evaluation of the vestibular system. This study adds to a small body of literature existing regarding vestibular function in CI candidates and highlights the prevalence of underlying vestibular dysfunction in this patient population. Moreover, this data demonstrates the significant portion of patients who have preexisting bilateral, possibly subclinical, vestibular weakness. We found that more than 10% of patients who undergo cochlear implant evaluation will exhibit some degree of bilateral weakness preoperatively. This is significant as patients with underlying bilateral weakness may have difficulty compensating for further iatrogenic vestibular insult as a result of surgery and may be at risk for increased postoperative dizziness and potential longer lasting detrimental effects of quality of life. The health of a patient’s vestibular system is important information to consider in the surgical planning and preoperative counseling of patients who are undergoing CI.

Bilateral vestibular dysfunction is a condition with the potential to significantly impact an individual’s quality of life and ability to function in society. A systematic review of symptoms of bilateral vestibulopathy by Lucieer et al (7) noted that patients afflicted by this condition suffer not only from imbalance (86–91%) and oscillopsia (50–70%) but may also experience extra-vestibular symptoms such as depression, impairments in memory and concentration, and social isolation (5). A study by Ward et al (8), investigating the functional impact of bilateral vestibular hypofunction (BVH) on US adults, reported that 44% of adults suffering from BVH changed their driving habits due to symptoms, 56% reduced their participation in social events, and 58% had reported difficulties performing activities of daily living. Adults with BVH also had a 31-fold increase in the odds of fallings and 25% reported a recent fall-related injury signifying the detrimental effect on the quality of life for those afflicted with BVH (9).

Nayak et al (3) similarly evaluated cochlear implant candidates with preoperative VNG and found that of 149 patients, 21.5% had evidence of BVH. The authors noted that patients with an abnormal preoperative VNG (unilateral and bilateral hypofunction) were more likely to experience postoperative dizziness lasting greater than 1 month. Specifically, 78.6% of dizzy patients had evidence of preexisting vestibular weakness. However, they did not find a significant difference in the rates of long-lasting postoperative dizziness in patients with unilateral versus bilateral findings on preoperative VNG (6).

The aim of this study was to identify prognostic indicators and risk factors for underlying bilateral vestibular dysfunction in cochlear implant candidates to help determine which patients may benefit from further vestibular workup before surgery. This is the first study, to our knowledge, to explore patient factors associated with bilateral vestibular dysfunction in the population of cochlear implant candidates. None of the medical comorbidities included for analysis were found to be associated with a risk for underlying bilateral vestibular weakness. However, we found that patients with bilateral vestibular dysfunction had a significantly higher BMI than those with normal vestibular function or unilateral vestibular dysfunction. While not quite reaching statistical significance, patients with bilateral vestibular weakness tended to be younger (Table 1). This may indicate a more aggressive pathology resulting in damage to both the cochlea and vestibular systems, as well as leading to hearing loss at a younger age with loss of residual hearing in the lower tones. Further investigation is needed to explore this relationship.

The severity of hearing loss at the low frequencies (250, 500, and 1000 Hz) was found to correlate with bilateral vestibular dysfunction. Moreover, we were able to define a cutoff point of hearing loss of 60 dB or worse at 250 Hz as an indicator for higher risk of bilateral weakness, with 100% sensitivity and maximized specificity of 34.5% when completing caloric testing for patients with that hearing cutoff. As all patients included in this study are CI candidates, all have severe hearing loss from 1000 Hz and above but may have preserved or residual hearing at the lower frequencies. The loss of hearing in the low frequencies indicates more extensive damage to the cochlea and may, based on the findings of this study, indicate more damage to the vestibular system as well. Given the potential for increased risk for further vestibular insult and damage after CI, there is a theoretical increased risk for patients with underlying asymptomatic vestibular dysfunction, particularly bilateral dysfunction, to develop a worse and possibly symptomatic bilateral dysfunction that could impair function and quality of life. We advocate for the use of preoperative VNG in all patients undergoing workup for CI, but particularly for those reaching the audiologic cutoff of hearing 60 dB at 250 Hz, as this captures all patients with existing bilateral dysfunction. There is little downside to performing preoperative VNG as potential adverse effects are temporary patient discomfort and added time and cost. Adding vestibular testing to the battery of testing performed as part of the cochlear implant evaluation will allow us to incorporate this data in surgical planning, patient counseling, and optimization of the patient’s balance if needed before surgery.

Limitations of this study include the lack of postoperative outcome data. Further investigation of postoperative dizziness and correlation with preoperative VNG findings are needed. Due to the retrospective nature of this study, it is difficult to know if patients with bilateral vestibular weakness had any overt or subclinical symptoms correlating to their VNG findings. Further prospective studies are needed to better understand the relationship between preoperative VNG findings and patient-reported symptoms of vestibular dysfunction.

## CONCLUSIONS

More than one-third of traditional CI candidates have some degree of underlying vestibular dysfunction and 10.5% exhibit a preexisting bilateral weakness. The results of this study indicate that audiologic data may be a useful prognostic indicator of preexisting bilateral vestibular weakness, with a pure tone threshold of 60 dB or worse at 250 Hz, in the worse hearing ear, as a cutoff point to identify these patients with 100% sensitivity and 34.5% specificity. Given the well-documented detrimental effects of bilateral vestibular weakness on quality of life, we recommend that all patients who meet this audiologic cutoff undergo vestibular testing to assess for an underlying weakness preoperatively and that this information be considered in surgical planning, counseling, and consideration of optimization with vestibular physical therapy.

## FUNDING SOURCES

None declared.

## CONFLICT OF INTEREST

None declared.

## References

[1] Guan R Wang Y Wu S. Vestibular function in children and adults before and after unilateral or sequential bilateral cochlear implantation. Front Neurol. 2021;12:675502. 33995266 10.3389/fneur.2021.675502 PMC8116579

[2] Gupta A Raj P. Compensated vestibular dysfunction post cochlear implantation in children with sensorineural hearing loss: a prospective study. Indian J Otolaryngol Head Neck Surg. 2018;70:200–204. 29977841 10.1007/s12070-017-1054-0 PMC6015564

[3] Nayak N Kellermeyer B Dornton L Heyd C Kim CS Wazen JJ. Vestibular dysfunction in cochlear implant candidates: prevalence and outcomes. Am J Otolaryngol. 2022;43:103171. 34509078 10.1016/j.amjoto.2021.103171

[4] Rasmussen KMB West N Tian L Cayé-Thomasen P. Long-term vestibular outcomes in cochlear implant recipients. Front Neurol. 2021;12:686681. 34456848 10.3389/fneur.2021.686681 PMC8385200

[5] Wang R Chao X Luo J. Objective vestibular function changes in children following cochlear implantation. J Vestib Res. 2022;32:29–37. 34633335 10.3233/VES-190763 PMC9249293

[6] West N Klokker M Cayé-Thomasen P. Vestibular screening before cochlear implantation: clinical implications and challenges in 409 cochlear implant recipients. Otol Neurotol. 2021;42:e137–e144. 33229879 10.1097/MAO.0000000000002898

[7] Lucieer F Duijn S Van Rompaey V. Full spectrum of reported symptoms of bilateral vestibulopathy needs further investigation-a systematic review. Front Neurol. 2018;9:352. 29915554 10.3389/fneur.2018.00352 PMC5994412

[8] Ward BK Agrawal Y Hoffman HJ Carey JP Della Santina CC. Prevalence and impact of bilateral vestibular hypofunction: results from the 2008 US National Health Interview Survey. JAMA Otolaryngol Head Neck Surg. 2013;139:803–810. 23949355 10.1001/jamaoto.2013.3913 PMC4839981

[9] Jacobson GP Shepard NT. Balance Function Assessment and Management. Plural Publishing; 2008.

[10] Hansen S Anthonsen K Stangerup SE Jensen JH Thomsen J Cayé-Thomasen P. Unexpected findings and surgical complications in 505 consecutive cochlear implantations: a proposal for reporting consensus. Acta Otolaryngol. 2010;130:540–549. 19958250 10.3109/00016480903358261

